# 

**Published:** 1876-10

**Authors:** 


*CONTENTS.*

| | Page |
|---|---|
| Causes of Fever and Ague | 419 |
| Poisonous Soap | 421 |
| Furnaces | 422 |
| Pure Water | 423 |
| Monomaniacs | 425 |
| Variety in Diet | 426 |
| Wholesome Flour | 429 |
| Wheat and Flour | 433 |
| The Health Food Company | 434 |
| An Erring Witness | 439 |
| A Bit of Humor | 440 |
| A New Enterprise | 441 |
| "Fire on the Hearth" | 442 |
| Dr. Dewey's Enterprise | 442 |
| Pertaining to Insurance | 443 |
| Book Reviews | 444 |
| Notice to Dyspeptics | 444 |
| Roserl—Concluded | 445 |
| Bowel Complaints of Children | 454 |

